# Green Synthesis of Zinc Oxide Nanoparticles Using an Aqueous Extract of *Punica granatum* for Antimicrobial and Catalytic Activity

**DOI:** 10.3390/jfb14040205

**Published:** 2023-04-07

**Authors:** Amr Fouda, Ebrahim Saied, Ahmed M. Eid, Fayza Kouadri, Ahmed M. Alemam, Mohammed F. Hamza, Maha Alharbi, Amr Elkelish, Saad El-Din Hassan

**Affiliations:** 1 Department of Botany and Microbiology, Faculty of Science, Al-Azhar University, Nasr City, Cairo 11884, Egypt; 2 Faculty of Pharmacy, Middle East University, Amman 11831, Jordan; 3 School of Nuclear Science and Technology, University of South China, Hengyang 421001, China; 4 Nuclear Materials Authority, P.O. Box 530, El-Maadi, Cairo 11728, Egypt; 5 Department of Biology, College of Science, Princess Nourah Bint Abdulrahman University, P.O. Box 84428, Riyadh 11671, Saudi Arabia; 6 Biology Department, College of Science, Imam Mohammad ibn Saud Islamic University (IMSIU), P.O. Box 90950, Riyadh 11623, Saudi Arabia; 7 Botany and Microbiology Department, Faculty of Science, Suez Canal University, Ismailia 41522, Egypt

**Keywords:** plant-based ZnO-NPs, *Punica granatum*, free radicals, ROS, antimicrobial activity, photocatalysis

## Abstract

The peel aqueous extract of *Punica granatum* was utilized to fabricate zinc oxide nanoparticles (ZnO-NPs) as a green approach. The synthesized NPs were characterized by UV-Vis spectroscopy, Fourier transform infrared (FT-IR), X-ray diffraction (XRD), transmission electron microscopy (TEM), and scanning electron microscopy, which was attached to an energy dispersive X-ray (SEM-EDX). Spherical, well arranged, and crystallographic structures of ZnO-NPs were formed with sizes of 10–45 nm. The biological activities of ZnO-NPs, including antimicrobial and catalytic activity for methylene blue dye, were assessed. Data analysis showed that the antimicrobial activity against pathogenic Gram-positive and Gram-negative bacteria, as well as unicellular fungi, was observed to occur in a dose-dependent manner, displaying varied inhibition zones and low minimum inhibitory concentration (MIC) values in the ranges of 6.25–12.5 µg mL^–1^. The degradation efficacy of methylene blue (MB) using ZnO-NPs is dependent on nano-catalyst concentration, contact time, and incubation condition (UV-light emission). The maximum MB degradation percentages of 93.4 ± 0.2% was attained at 20 µg mL^−1^ after 210 min in presence of UV-light. Data analysis showed that there is no significant difference between the degradation percentages after 210, 1440, and 1800 min. Moreover, the nano-catalyst showed high stability and efficacy to degrade MB for five cycles with decreasing values of 4%. Overall, *P. granatum*-based ZnO-NPs are promising tools to inhibit the growth of pathogenic microbes and degradation of MB in the presence of UV-light emission.

## 1. Introduction

The creation of multifunctional nanomaterials for implementation in numerous industries is currently a key issue for researchers due to their unique properties. Engineering, biomedicine, environmental clean-up (environmental remediation), and bioenergy have been considered the main fields for nanomaterial applications [1,2]. Moreover, the environmentally safe techniques have been preferred over the chemical and physical ones when creating NPs, especially when employing them to inhibit the growth of pathogenic microbes and to remediate environmental wastes [2]. The size of nanoparticles (1–100 nm) has a critical role in their applications, and the activity was increased at decreasing sizes due to exceptional characteristics, including increased mechanical capabilities, high catalytic activity, distinctive optical features, and low melting point [3,4]. Recently, a novel advancement in the use of metals and metal oxide nanoparticles in many applications was developed [2]. Zinc oxide nanoparticles (ZnO-NPs) are one of several metal oxide-NPs utilized in bioremediation and antimicrobial applications. ZnO is a n-type semiconductor with a range of additional advantageous properties, including being non-toxic, exhibiting a wide band gap, high binding energy, and good chemical and thermal stability at ambient temperature [5]. Various chemical and mechanical procedures, such as mechanochemical methods, hydrothermal methods, and the sol-gel method, have been conventionally used on a large scale for obtaining ZnO-NPs. The effectiveness of the commercial sector is often shown to have a wide range of limitations by these methods. For instance, the chemical production of NPs uses poisonous and harmful compounds as reductants and stabilizers, while the high temperature pyrometallurgical method uses high energy [6,7]. Consequently, this makes people aware of the environment’s state, and it makes them motivated to find out more about the one-pot green synthesis process. 

The advantages of green strategy, including its simplicity, eco-friendliness, and low operating costs, have made ZnO-NP fabrication more prevalent. Additionally, the green synthesis process uses safe solvents, including water and ethanol, achieving the objective of green chemistry [8,9]. In green methods, the toxic chemicals are replaced with a variety of renewable natural sources, such as plant extracts and biomass filtrate of different microorganisms (bacteria, fungi, yeast, actinomycetes, and microalgae) to increase the biosafety of ZnO-NPs [10,11]. These natural sources are rich in bioactive components, such as amino acids, carbohydrates, and polyphenols, which promote the formation of ZnO-NPs. Recently, a new procedure trend has emerged in the use of plant extracts to fabricate ZnO-NPs, such as aqueous extract of *Salvia hispanica*, *Abelmoschus esculentus*, orange fruit peel extract, *Deverra tortuosa*, *Malva Parviflora*, and *Phoenix dactylifera* waste, where the plant extracts act as capping and stabilizing agents, and they prevent the agglomeration of the particles [5,12,13]. 

The existence of harmful microorganisms and their activities is a prominent issues [14]. With the development of chemical antibiotics and their use in the treatment of diseases, bacteria have been attempting to develop antibiotic resistance. Additionally, the excessive use of chemical antibiotics has resulted in various environmental issues due to their adverse effects. [15]. Antibiotic efficiency is reduced because the majority of harmful microorganisms develop antibiotic resistance. According to studies, only a small percentage of pathogenic bacteria are resistant to all antibiotics, and more than 70% of them are resistant to at least one major class of chemical antibiotic [16,17]. Due to harmful bacteria’s resistance to typical bactericidal agents found in the environment, it is imperative to create new, inexpensive, and environmentally acceptable bactericidal agents. These compounds introduce new prospects for nanoparticles with bactericidal characteristics. Utilizing metal and metal oxide nanoparticles and nanocomposites is one of the beneficial strategies used to combat bacterial activity. This class of bactericidal components have gained a lot of interest due to their small particle size, increased surface area, and enhanced bactericidal action [18]. ZnO-NPs are among metal oxide-NPs that are characterized by their high bactericidal properties [19]. 

For the past few decades, water pollution has been considered a huge issue for humanity. The World Bank estimates that 17–20% of water pollution is caused by the dyeing and finishing of textiles, which generates the largest group of hazardous and organic chemicals [20]. The main cause of environmental pollution comes from the usage of organic dyes in the food and textile industries. In addition, these contaminants are too hazardous, causing cancer in humans and harming the environment [21,22]. For instance, tiny quantities of an organic dye of methylene blue (MB) in water can cause a variety of illnesses, including anemia due to hemolysis, irritations, and stomach problems. Moreover, MB dye is characterized as being non-biodegradable, highly toxicity, carcinogenic, and it causes severe negative effects when it is disposable in an environment without treatment [23]. Among the negative impacts of MB on human health are causing abdominal, mental, digestive, and respiratory disorders, as well as blindness, diarrhea, nausea, cyanosis, vomiting, gastritis, methemoglobinemia, jaundice, tissue necrosis, increased heart rate, and skin itching and redness [23,24]. However, the use of this dye is crucial to the industry’s production of textile goods, particularly when dyeing fabrics, pharmaceuticals, medicine, printing, the food industry, and paints [25,26]. There are several methods for detoxification, including distillation, coagulation and flocculation, ultrafiltration, microfiltration, UV therapy, and reverse osmosis, among others, which are being used, but a more suitable procedure to clean up the dye-polluted water is still required [27]. Consequently, it is thought that semiconductor-based photocatalysis is the most dependable and safe approach. ZnO-NPs have received a lot of attention because of their chemical stability, non-toxicity, thermal stability, and photocatalytic ability when compared to other semiconductors, as well as their higher decomposition rates for organic pollutants due to their higher quantum efficiency. Because ZnO NPs are harder substances, they do not suffer from dislocation degradation throughout the procedure [28]. Recently, ZnO-NPs fabricated by an aqueous extract of lemon peel were used for the degradation of antibiotic ciprofloxacin under UV-light irradiation (photocatalysis) [29].

The photocatalysis technique means enhancement of the activity of catalyst in the presence of light sources. ZnO-NPs are unique substances that have large surface area and a broadband gap with a binding energy of 3.37 eV, and they are exceptionally reliable and increase photocatalytic efficiency [5]. The light beam from different sources, such as halogen light, sunlight, or UV, is adsorbed on the surface of the nano-catalyst, creating electron–hole pairs, followed by reaction with dissolved substances in the solution to generate free radicals, such as hydrogen peroxide, hydroxyl radicals, and superoxide anions [30,31]. The activity of nano-catalysts in photocatalytic reactions is dependent on their potential to generate electron–hole pairs and free radicals, which attack the pollutants and degrade them into non-toxic substances. Additionally, the high bactericidal activity of ZnO-NPs in the presence of light emission could be related to the production of free radicals that enhance the reactive oxygen species (ROS), and this process can lead to cell death [32]. The creation of ZnO-NPs, utilizing an aqueous extract of plant waste (the peel of *Punica granatum*) for dual function, antimicrobial activity, and dye removal, is what distinguishes this current study from previous ones. 

Therefore, the main hypothesis of the current study was that ZnO-NPs were synthesized using a green approach and utilized to inhibit the growth of pathogenic microbes and the degradation of MB dye. To achieve this hypothesis, the ZnO-NPs were fabricated by the activity of aqueous peel extract of *Punica granatum* and characterized by UV-Vis spectroscopy, the Fourier transform infrared method (FT-IR), X-ray diffraction (XRD), transmission electron microscopy (TEM), and scanning electron microscopy connected to an energy dispersive X-ray (SEM-EDX). The antimicrobial activity of plant-based ZnO-NPs against pathogenic Gram-positive, Gram-negative bacteria, and unicellular fungi was investigated. Additionally, the photocatalytic activity of phyto-synthesized ZnO-NPs against methylene blue dye was studied. 

## 2. Materials and Methods

### 2.1. Materials

Zinc acetate (Zn(CH_3_COO)_2_‧2H_2_O) was used as a precursor for ZnO-NPs, and it was purchased from Sigma-Aldrich (Cairo, Egypt), possessing a purity percentage of 99%. The components of media used in the current study, as well as methylene blue dye, were obtained from Sigma-Aldrich (Taufkirchen, Germany), with purity percentages in the ranges of 95–99%. All reactions were achieved using distilled water (dH_2_O). The bacterial and fungal strains used in antimicrobial activity were obtained from the Microbial Physiology Lab., Faculty of Science, Al-Azhar University, Cairo, Egypt. 

### 2.2. Biogenic Synthesis of ZnO-NPs Using Punica granatum

#### 2.2.1. Prepared Peel Aqueous Extract

The peels of *P. granatum* were collected and washed thrice with distilled H_2_O to remove any adhering debris, and then they were left to dry at 40 °C and ground to form powder. Five grams of prepared powder were mixed with 100 mL dH_2_O, and then they were heated for 60 min at 55 °C, using a magnetic stirrer (rpm = 120). Finally, the mixture was filtered, followed by centrifugation at 6000 rpm for 10 min. The supernatant was collected and used as a reducing agent to form ZnO-NPs.

#### 2.2.2. Peel Aqueous Extract-Based Biogenic Synthesis of ZnO-NPs

To achieve a final concentration of 5 mM, 10 mL of *P. granatum* peel aqueous extract was added to 90 mL of dH2O that contained the metal precursor (Zn(CH_3_COO)_2_‧2H_2_O). Drops of 1N NaOH were added to the mixture, and it was then stirred at 40 °C for an hour to bring the pH level down to 8.0. The combination was then incubated overnight at room temperature and in the dark. ZnO-NPs are formed when a yellowish-white precipitate appears [11]. The precipitate was separated using centrifugation, rinsed three times with deionized water, and dried in an oven for three hours at 200 °C [33]. 

### 2.3. Characterizations

The phyto-synthesized ZnO-NPs were first characterized by UV-Vis spectroscopy (JENWAY-6305, Staffordshire, UK). Approximately 2 mL of formed yellowish-white solution was added to the cuvette, and the spectra were measured in the range of 200–800 nm to detect the maximum surface plasmon resonance (SPR). X-ray diffraction is a useful technique for giving more information about the crystallinity or amorphous properties of plant-synthesized ZnO-NPs. The XRD spectra were investigated at 2θ values in the range of 10°–80° using X’ Pert Pro (Philips, Eindhoven, the Netherlands). The apparatus conditions were Cu used as a X-ray source at λ = 1.54 Å and 30 mA and 40 KV [34].

The average crystal size was calculated based on XRD analysis using a Debye-Scherrer equation, as follows:

(1) Average crystalite size=Kλ/βcosθ

where K = 0.9, λ = 1.54 Å, and β is the full width at a half maximum in 2θ value. 

The Fourier transform infrared (FT-IR) spectra of phyto-synthesized ZnO-NPs were identified using a Cary 630 FTIR spectrometer (Tokyo, Japan). Moreover, the bending and stretching vibration bands, as well as the functional groups in the peel aqueous extract of *P. granatum,* were detected using FT-IR. In this method, 300 mg of synthesized NPs was mixed with KBr and hard-pressed to form a disk before being scanned in the ranges of 400–4000 cm^−1^. Additionally, several drops of plant aqueous extract were loaded onto a KBr disc, followed by scanning at the same wavenumber [35]. 

Transmission electron microscopy (TEM JEOL 1010, Tokyo, Japan) was used to examine the size, shape, and aggregation of phytosynthesized ZnO-NPs. Approximately, 4 mg of synthetic ZnO-NPs were sonicated for 15 min while suspended in 2 mL of ethanol. A few drops of the preceding suspension were loaded on a TEM grid (C-coated Cu-grid), where they were let to dry for about five minutes before being analyzed [11]. The elemental compositions of synthesized metal oxide nanoparticles were detected using a scanning electron microscope attached to an energy dispersive X-ray unit (SEM-EDX). The JEOL-JSM 6360 (JEOL-Ltd., Tokyo, Japan) was adjusted at an accelerating voltage of 200Kv and connected to an EDX (Thermo-Fisher Scientific, Madison, Waltham, MA, USA) apparatus. A few drops of the suspended solution were dropped on a holder and coated with gold by a sputter coater in a vacuum [36]. 

Finally, dynamic light scattering (DLS) was used to determine the size, size distribution, and polydispersity index (PDI) of green-produced ZnO-NPs (DLS). To prevent the release of extra peaks or shadows during scattering, the synthesized ZnO-NPs were dissolved in Milli-Q H2O (high pure H_2_O) in this analysis. Additionally, after being dispersed in Milli-Q H_2_O, the charge of the NP’s surface was examined using a zetasizer (Nano-ZS, Malvern, United Kingdom) [37]. 

### 2.4. Antimicrobial Activity

The activity of plant-based synthesized ZnO-NPs to inhibit the growth of pathogenic bacteria and unicellular fungi was investigated. The following strains were used to assess the antimicrobial activity, *Staphylococcus aureus* ATCC6538, *Bacillus subtilis* ATCC6633 (as Gram-positive bacteria), *Pseudomonas aeruginosa* ATCC9022, *Escherichia coli* ATCC8739 (as Gram-negative bacteria), and *Candida albicans* ATCC10231 (as unicellular fungi) by the agar well diffusion method. In this method, the selected bacterial strains were sub-cultured on Muller Hinton agar media (ready prepared oxide), whereas the unicellular fungus was sub-cultured on Sabouraud agar media (Ready Prepared-Oxide) for 24 h at 35 °C. After that, re-streaking of each strain was performed on a new plate by sterilized swab, and wells (0.6 mm) were made by sterilized cork borer and filled with 100 µL of prepared ZnO-NPs solution (200 µg mL^−1^), and they were incubated at 35 ± 2 °C for 24 h. At the end of the incubation period, the results were recorded by the diameter of the clear zone around the well (by mm). Several ZnO-NPs concentrations (100, 50, 25, 12.5, 6.25, and 3.13 µg mL^−1^) were prepared and checked for their activity, in the same manner, to detect the minimum inhibitory concentration (MIC). 

### 2.5. Catalytic Activity against Methylene Blue (MB) Dye

The efficacy of plant-based ZnO-NPs to degrade MB as a dye model was investigated in the presence of UV-light (mercury lamp, λ > 253 nm). All of the ZnO-NPs concentrations (5, 10, 15, and 20 µg mL^−1^) were added to MB solution (10 mg L^−1^) for different contact times (10, 30, 60, 90, 120, 150, 180, 210, 1440, and 1800 min). Before the experiment, the MB solution was subjected to stirring at room temperature for 30 min. to reach the absorption/desorption equilibrium between MB and the surface of the nano-catalyst [19]. By combining the nano-catalyst and the MB in light conditions with air bubble aeration, the effectiveness of each ZnO-NPs concentration to adsorb or degrade the MB was examined in comparison to the control (MB solution without ZnO-NPs). Approximately, 2 mL of each treatment was withdrawn at a regular interval time, and centrifuged at 1000 rpm for 10 min. Clear supernatant was collected. Finally, their optical density was read at a maximum λ_max_ of MB at 664 nm using the M-ETCAL spectrophotometer. The percentages of color removal were calculated by the following equation:

(2) $$\mathrm{Decolorization}\;\mathrm{percentages}\;(\%)=\frac{A-B}{A}\times 100$$

where A is the initial absorbance, and B is the final absorbance at interval times. 

The activity of different reactive species, including hydroxyl radicals (^•^OH), superoxide radicals (^•^O_2_^–^), and holes (h^+^) into MB degradation, was investigated by the trapping method. In this method, 1 mM of isopropyl alcohol (IPA), 1 mM benzoquinone (QB), and 1 mM of ethylenediaminetetraacetate (EDTA) were added to a reaction solution under optimum conditions (20 µg mL^−1^ of green synthesized ZnO-NPs to MB solution (10 mg L^−1^) for 210 min under UV-light irradiation at pH 7). These reagents act as quenchers for ^•^OH, ^•^O_2_^–^, and h^+^, respectively [29]. At the end of contact time, the absorbance of the mixture was measured as 664 nm, followed by detecting the degradation percentages, as mentioned above. 

To further support the adsorption of dye on the surface of the catalyst, the nano-catalyst was gathered at ideal catalytic conditions and examined using SEM-EDX and FT-IR. ZnO-NPs produced through phytosynthesis underwent a reusability test that was evaluated across five cycles. After being collected from the first cycle, the nano-catalyst was washed three times with distilled H_2_O and dried in an oven at 50 °C to remove any remaining water before being utilized in the second cycle. 

### 2.6. Statistical Analysis

Data collected in the present study are presented as the means of three independent replicates and subjected to statistical analysis using the statistical package SPSS v17. The mean difference comparison between the treatments was analyzed by t-test or the analysis of variance (ANOVA), and it was subsequently analyzed by the Tukey HSD test at *p* < 0.05.

## 3. Results and Discussion

### 3.1. Punica Granatum-Based Green Synthesis of ZnO-NPs

**Figure 1:**
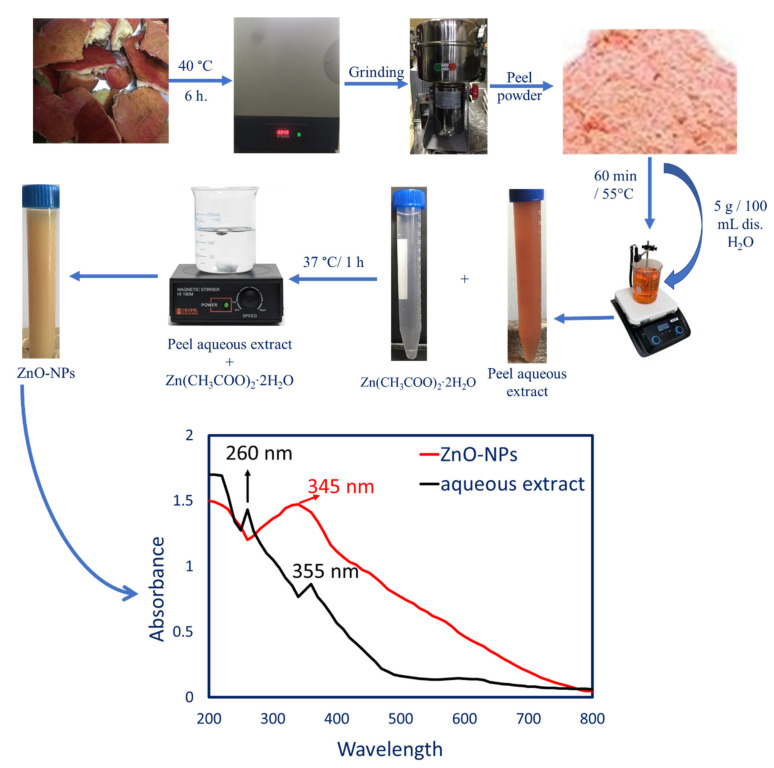
Green synthesis of ZnO-NPs using aqueous extract of *P. granatum,* showing the color change after mixing with metal precursor and measuring the UV-Vis spectroscopy for aqueous extract and plant-based ZnO-NPs to detect the maximum SPR peaks.

Recently, to prevent the detrimental effects of chemical and physical procedures on consumers and the ecosystem, green fabrications of nanomaterials, using various biological entities, such as bacteria, fungi, actinomycetes, algae, and plants, are receiving more attention [38]. Among green methods, phyto-fabrication is a promising approach for the synthesis of nanomaterials using plant extracts. This phenomenon is promising due to its simplicity, rapidness, low cost, high yield, biocompatibility, ease of collection of the final product, the ability to avoid the pathogenesis that can be released from the utilization of microorganisms, and varied plant metabolites that enable one to produce various shapes, as well as sizes with high stability nanoparticles [39]. In the current study, the aqueous extract of *P. granatum* peel was used as a catalyst to reduce Zn(CH_3_COO)_2_‧2H_2_O to form ZnO-NPs (Figure 1). The prospective mechanism of phyto-fabrication of ZnO-NPs, using *P. granatum,* is due to the presence of polyphenols, such as flavonoids, tannins, anthocyanidins, and phenolic acids that react with Zn^+2^ and form Zn(OH)_2_ by reducing and capping processes, followed by synthesis of ZnO-NPs by annealing treatment [40]. The presence of these polyphenolic compounds was confirmed by UV-Vis spectroscopy and FT-IR of peel aqueous extract. As shown, the UV chart of peel aqueous extract (Figure 1) displayed the presence of maximum absorbance (λ_max_) at 260 and 355 nm, signifying the presence of tannic acids and flavonoid compounds, as reported previously [41]. According to some researchers, the formation of ZnO-NPs can result from the reduction of Zn^+2^ by various metabolites in the aqueous extract to form zinc metal (Zn^0^) that reacts with dissolved oxygen to form ZnO, followed by capping with active metabolites to prevent agglomeration and to increase the NPs’ stability [39,42]. 

### 3.2. Characterizations

#### 3.2.1. Color Change and UV-Vis Spectroscopy

A color change from red to yellowish white is the first indicator for the plant-based biogenic production of ZnO-NPs utilizing *P. granatum* peel aqueous extract. The color change was carried out gradually, indicating the reduction of the metal precursor by plant metabolites to nanostructures. In the current study, the maximum color change was achieved after 24 h, and this change can be attributed to the excitation of plasmon vibrations on the surface of metal and metal oxide-NPs. The maximum absorbance that refers to the maximum SPR specific for ZnO-NPs was detected using UV-Vis spectroscopy. SPR peaks originated in the UV region because of the collective oscillation of resonant electrons in the conduction band along the electromagnetic field [43,44]. The maximum intensity of SPR peaks, as well as their width, is dependent on NPs’ shape, size, and temperature [45]. Herein, the maximum SPR of phyto-fabricated ZnO-NPs was observed at 345 nm (Figure 1). The obtained absorption peak is related to the range of light absorption of ZnO-NPs at a wavelength of 350–380 nm. In a recent study, the maximum SPR band of ZnO-NPs, fabricated by flower extract of *P. granatum,* was noticed at 345 nm, whereas those synthesized by the action of leaves of *P. granatum* aqueous extract showed two peaks at 284 nm and 357 nm [11]. 

#### 3.2.2. X-ray Diffraction

**Figure 2:**
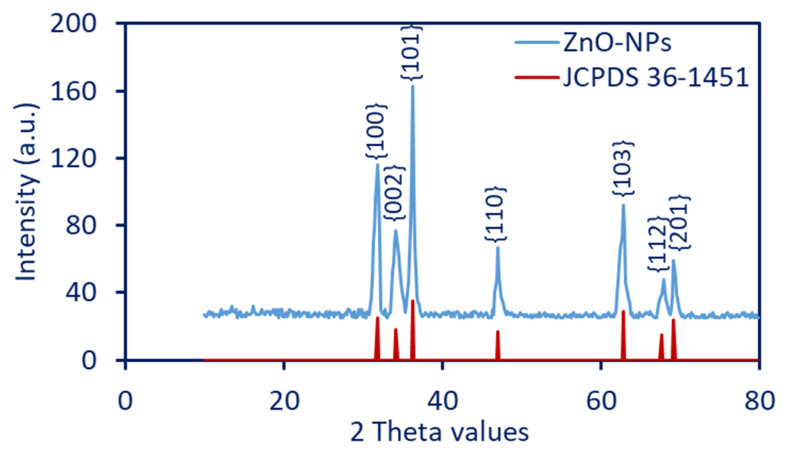
X-ray diffraction of phyto-synthesized ZnO-NPs showed the diffraction peaks at varied 2 theta values compared to the JCPDS card number of 36–1451, which confirms a crystalline nature.

The powder form of phyto-synthesized ZnO-NPs has undergone the X-ray diffraction analysis in the ranges of two theta values of 10–80°, as shown in Figure 2. The XRD reveals the crystallographic structure of synthesized NPs, according to the standard of the Joint Committee on Powder Diffraction (JCPDS). As shown, the prominent diffraction peaks for ZnO-NPs were observed at two theta values of 31.7°, 34.4°, 36.2°, 47.5°, 56.6°, 62.8°, 67.9°, and 69.2°, which were indexed to crystal planes of {100}, {002}, {101}, {102}, {110}, {103}, {112}, and {201}, respectively. The XRD chart revealed the crystalline nature of phyto-synthesized ZnO-NPs, as compared to the JCPDS card number 36–1451 [46]. The obtained data are compatible with various studies that reveal the crystalline nature of ZnO-NPs fabricated by plant extract, according to XRD diffraction peaks at the same two theta values [11,39]. The absence of extra peaks in the XRD chart confirmed the high purity of plant-based ZnO-NPs. The broad bases of Bragg’s XRD peaks reveal that the sizes of synthesized NPs are small, as reported previously [47]. In the current study, the crystallite size of ZnO-NPs was calculated using the Debye-Scherrer equation. The measurement was achieved by using the maximum diffraction peak of {101} that originated at a 2° value of 36.2°. The analysis showed that the crystallite size was 43 nm. 

#### 3.2.3. Fourier Transform Infrared (FT-IR)

**Figure 3:**
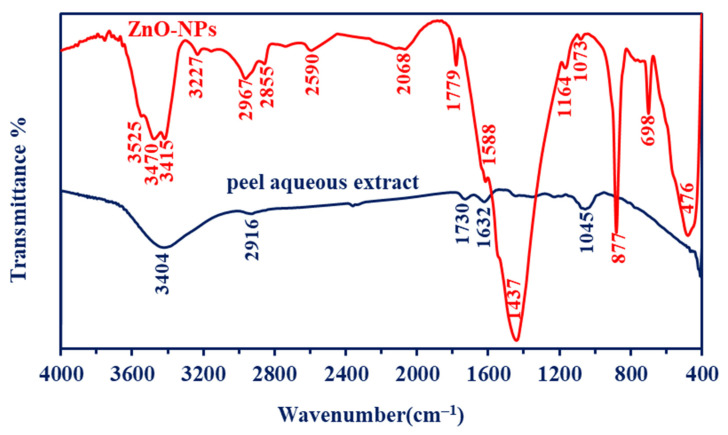
FT-IR for *P. granatum* aqueous extract and synthesized ZnO-NPs.

The produced final material, ZnO-NP, was fabricated using peel aqueous extract (known as the control), and it was analyzed by the FTIR, as shown in Figure 3. The peaks related to O-H overlapped with NH vibrations, and they appeared as a broad band at 3404 cm^−1^ [48], and this band appeared as Zn produced with different internal small bands at 3227, 3415, 3470, and 3525 cm^−1^. This confirms the production of different types of amines (primary and secondary) besides OH groups [49]. The C–H aliphatic appeared at 2916 for the control sample, vis, 2855 and 2967 cm^−1^ for the Zn material. A peak at 2590 cm^−1^ for ZnO material is related to thiol stretching bands that were not found in the control sample [50]. A peak at 2068 cm^−1^ (for the ZnO-NPs) is related to the sulfonic groups (from the medium) stretching bands. Peaks at 1730 cm^−1^ (for the control) and 1779 cm^−1^ (for the ZnO-NPs) are related to the ester group, while the control has NH stretching and C=O at 1632 cm^−1^ which is not found in the synthesized material. Other distinguished peaks for the ZnO-NPs sample appeared at 1437 cm^−1^ for the carboxylate ions and the organic sulfate groups, whereas the peak at 1588 cm^−1^ is related to the NH band of primary amines (with low intensity, which indicates that binging occurred with ZnO ions) and stretching C–C [11,51]. Peaks at 1045 cm^−1^ in the plant aqueous extract are related to C-O-C for C-N stretching and C-O-C, which is asymmetric. By contrast, the C-O-C, C-N stretching, and C-O-C asymmetry in ZnO-NPs appeared at 1164 and 1073 cm^−1^. The sharp band (not found in the control) at 877 cm^−1^ is related to C-O-S stretching [52]. The stretching band of zinc and oxygen is confirmed by the emergence of peaks in the range of 450–700 cm^−1^ (not present in the plant aqueous extract), which indicates that ZnO was successfully formed [11,46,53]. The presence of various functional groups related to different metabolites in plant aqueous extract demonstrated their function in ZnO-NPs reduction, capping, and stability. 

#### 3.2.4. TEM, SAED, and EDX

**Figure 4:**
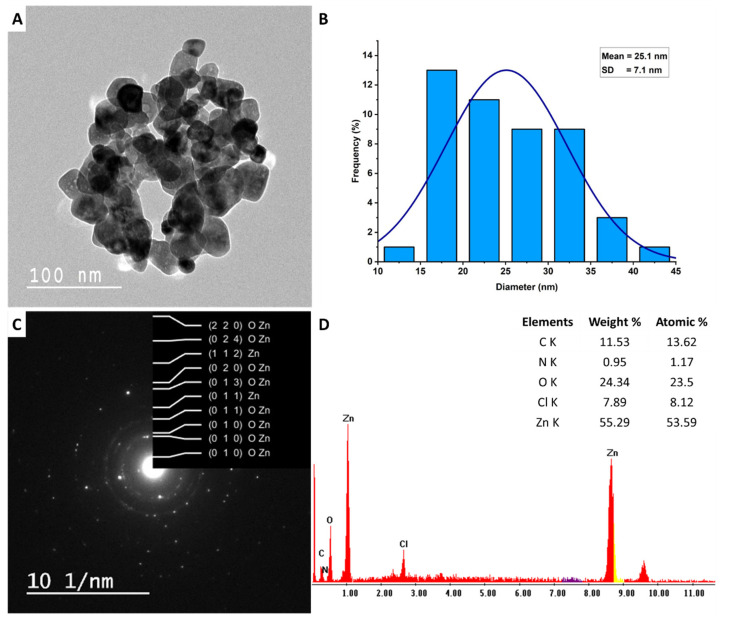
Characterization of phyto-synthesized ZnO-NPs. (**A**) TEM image; (**B**) size distribution; (**C**) SAED pattern; and (**D**) EDX analysis.

The TEM technique is widely used by researchers to examine the morphology, shape, size, and agglomeration of NPs. In the current study, the peel aqueous extract of *P. granatum* was effective in reducing zinc acetate to form spherical ZnO-NPs with little aggregation (Figure 4A). Moreover, the TEM image revealed that the size obtained ZnO-NPs had an average particle size of 25.1 nm and ranged in size from 10 to 45 nm (Figure 4B). The particle size from TEM analysis is compatible with the crystallite size obtained by XRD using the Debye-Scherrer equation. The results corroborated a study that reported that the size of ZnO-NPs produced by leaf aqueous extract of *P. granatum* acquired by TEM is close to those obtained by XRD analysis [46]. In contrast, the particle size of spherical ZnO-NPs fabricated by aqueous extract of peel *Citrus reticulata* peel obtained by TEM analysis was in the range of 23–90 nm, whereas the crystallite size obtained by XRD was 8.9 nm [54].

Several authors claimed that the size and shape of ZnO-NPs have a significant impact on their activity. For example, leaf aqueous extract of *Aloe vera* was used to fabricate cubic, hexagonal, cylindrical, and spherical ZnO-NPs [55]. The cubic shape showed promising antibacterial activity against *Staphylococcus aureus*, *Bacillus subtilis*, and *E. coli,* as compared with other synthesized shapes, whereas the spherical shape displayed high photocatalytic activity to remove methyl orange dye. Moreover, the antibacterial activity of ZnO-NPs against *Enterococcus faecalis, Streptococcus mutans,* and *Lactobacillus fermentum* varied according to their sizes [56]. The authors showed that the highest growth inhibition based on the formed clear zones was recorded for sizes 20 and 40 nm against *S. mutans*, as well as size 140 nm against *E. faecalis* and *S. mutans*. Interestingly, the smaller size (4–10 nm) of ZnO-NPs fabricated by extract of *Dysphania ambrosioides* showed high activity to inhibit the growth of pathogenic bacteria compared to larger sizes [57]. This phenomenon could be attributed to the high surface reactivity with small sizes and enhances their properties that allow for multiple uses [19].

The selected area electron diffraction (SAED) pattern of phyto-synthesized ZnO-NPs (Figure 4C) was indexed using CrysTBox ringGUI 1.16 by Miloslav Klinger (klinger at post dot cz) [58]. The SAED pattern reveals well defined circular rings associated with the planes of {220}, {024}, {112}, {020}, {013}, {011}, and {010}. These planes are indexed to the Wurtzite structure, which confirmed the polycrystalline nature of ZnO-NPs [59]. The chemical composition of as-formed ZnO-NPs was investigated using EDX analysis. The EDX analysis displayed clear absorption peaks for Zn at bending energies of 1 eV and 8.6 eV, as well as one absorption peak for oxygen at bending energy of 0.5 eV, indicating the successful formation of ZnO. As shown, the weight and atomic percentages of Zn and O were 55.29 and 24.34% and 53.59 and 23.5%, respectively, referring to the major component of the nanostructure as Zn and O (Figure 4D). In a similar study, the Zn and O represented the main components of ZnO-NPs fabricated by leaf aqueous extract of *Malva Parviflora* with the highest weight and atomic percentages [13]. On the other hand, the EDX chart reveals the presence of some weak signals for C, N, and Cl with weight and atomic percentages of 11.53, 0.95, and 7.89, as well as 13.62, 1.17, and 8.12, respectively. These weak absorption peaks could be due to the X-ray emission of capping agents, such as carbohydrates, proteins, and enzymes, which refer to their role in the reduction, capping, and stabilizing functions [60,61].

#### 3.2.5. DLS and Zeta Potential

**Figure 5:**
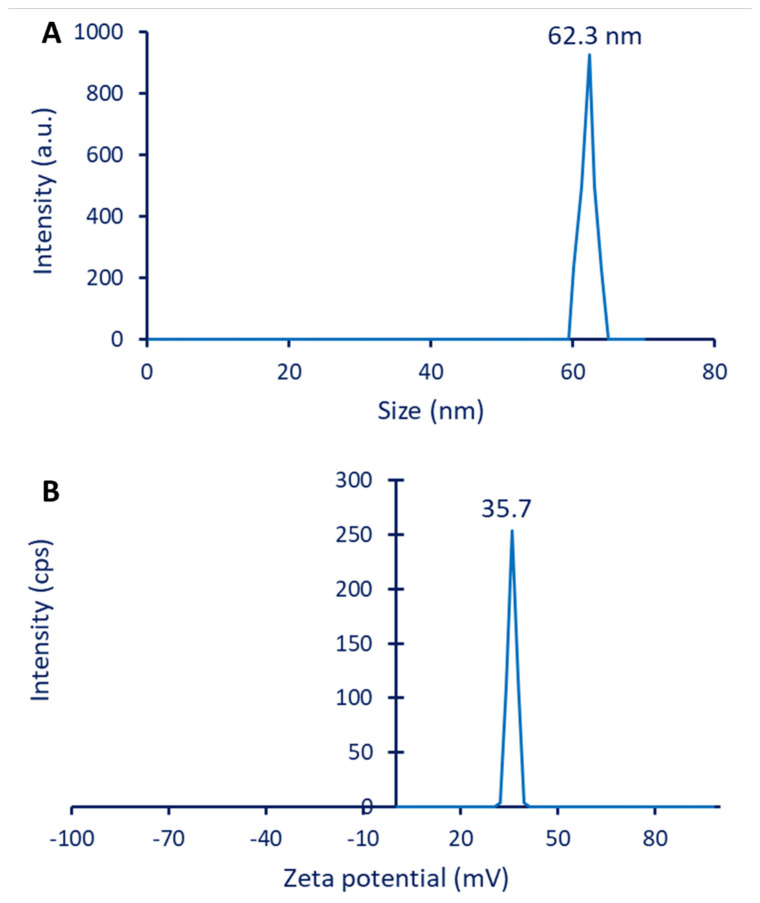
Dynamic light scattering (DLS) (**A**) and zeta potential value (**B**) of green synthesized ZnO-NPs.

Using DLS analysis, it was possible to determine the size and size distribution of the colloidal solution, as well as to detect the hydrodynamic residue of plant-synthesized ZnO-NPs. The analysis revealed that the average size of ZnO-NPs in the colloidal solution was 62.3 nm (Figure 5A), which is considered bigger than those obtained by TEM (25.1 nm) and XRD (43 nm). This finding may be explained by the method of measurement, which, in TEM, was achieved using dry particles, whereas DLS was performed in an aqueous solution, allowing the measurement of the hydrodynamic shell of the particles [62]. Additionally, the variation in sizes between different methods could be attributed to the DLS analysis, being affected by coating agents, non-homogenous distribution of ZnO-NPs in colloidal solution, and purity of solvent [62,63]. In a similar study, the average sizes of ZnO-NPs fabricated by leaf aqueous extract of *Pelargonium odoratissimum* were 34.1, 21.6, 14, and 76 nm, according to HR-TEM, FE-SEM, XRD, and DLS, respectively [64].

Moreover, the DLS analysis gives more predictions about the homogeneity of the sample in the aqueous solution through the measurement of the polydispersity index (PDI). When the PDI value is less than 0.4, the sample is deemed homogeneous. Homogeneity decreases for values between 0.4 and 1, and the sample is regarded as heterogeneous when the PDI value exceeds 1. [65]. The PDI value in the current study was 0.312, indicating good NP dispersion in an aqueous solution and high homogeneity.

The stability of ZnO-NPs was detected by measuring electrokinetic potential or Zeta potential (ζ-potential). This value originated due to the moving of particles in the solution under an electric force [66]. Herein, the ζ-potential value of green synthesized ZnO-NPs was 35.7 mV (Figure 5B). The stability of NPs, according to ζ-potential values, was designated as follows: highly unstable (ζ values in the ranges of ±0–10 mV), relatively stable (ζ values in the ranges of ±10–20 mV), stable (ζ values in the ranges of ±20–30 mV), and high stable (ζ values is higher than ±30 mV) [67]. This is based on obtained ζ value (35.7 mV), suggesting the high stability of synthesized ZnO-NPs. This stability could be attributed to the coating agents coming from plant extract, which increased the electrokinetic force [68]. Additionally, the presence of one positive charge on the surface of all ZnO-NPs in the colloidal solution enhances the repulsion between the particles and, hence, prevents aggregation, leading to an increase in stability [67]. 

### 3.3. Antimicrobial Activity

**Figure 6:**
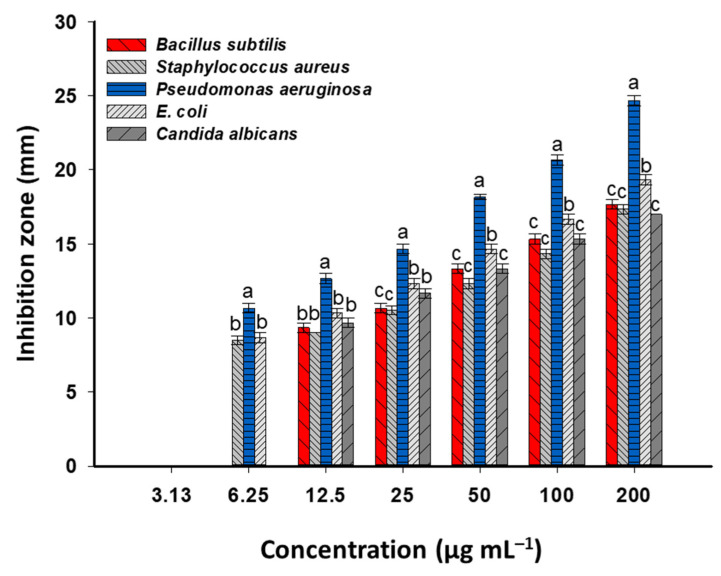
Antimicrobial activity of *P. granatum*-mediated green synthesis of ZnO-NPs against pathogenic microbes. The different letters at the same NPs concentration indicate that the data are statistically significant differences (*p ≤* 0.05, *n* = 3, error bars are ±SD).

The infectious diseases caused by different pathogenic bacteria and *Candida* are considered important global problems, particularly in developing countries. Moreover, arising from antibiotic-resistance strains, lack of appropriate vaccines, emergence of new mutants, and hospital-acquired infections, these developments increased the infection and outbreaks of pathogenic microbes. Thus, it is vital to produce new active biocompatible compounds that are economical, highly active, and secure for use by humans and ecosystems. ZnO-NPs are described as safe, and they have good biocompatibility with human cells, with promising antimicrobial activity [30]. ZnO-NPs are semiconductors that have band gaps in the visible region, which improve their properties; therefore, their utility in biomedical and biotechnological applications is increased by these characteristics. In the current study, the activity of phyto-synthesized ZnO-NPs against Gram-positive bacteria (*Bacillus subtilis* and *Staphylococcus aureus*), Gram-negative bacteria (*Pseudomonas aeruginosa* and *Escherichia coli*), and unicellular fungi (*Candida albicans*) were assessed (Figure 6). Analysis of variance showed that the antimicrobial activity of ZnO-NPs was dose-dependent, which is compatible with various published studies [19,69,70]. The maximum inhibition zones were obtained at high ZnO-NPs concentrations (200 µg mL^−1^) to be 17.7 ± 0.6, 17.3 ± 0.6, 24.7 ± 0.7, 19.3 ± 0.6, and 17.0 ± 0.1 mm for *B. subtilis*, *S. aureus*, *P. aeruginosa*, *E. coli*, and *C. albicans*, respectively (Figure 6). Similarly, the highest inhibition zones against *E. coli* (15.0 ± 0.0 mm), *Klebsiella pneumoniae* (13.3 ± 0.3 mm), *S. aureus* (12.7 ± 0.2 mm), *Streptococcus pneumoniae* (12.7 ± 0.2 mm), and *C. albicans* (12.7 ± 0.2 mm) were achieved by using 10 mg of ZnO-NPs, synthesized by leaf aqueous extract of *Parthenium hysterophorus*, which were compared to low concentrations (1, 3, and 5 mg) [71]. The antimicrobial activity of green synthesized nanomaterials is dependent on various factors, such as concentration, incubation condition, incubation time, and method of synthesis. For instance, the Se-NPs fabricated by endophytic *Penicillium crustosum* strain EP^−1^ showed varied antimicrobial activity based on incubation conditions (dark and light) [72]. The authors reported that the inhibition zones were increased from 15.3 ± 0.6 mm, 14.8 ± 0.5 mm, 14.3 ± 0.7 mm, and 14.7 ± 0.6 mm in dark conditions to 17.7 ± 0.6 mm, 16.0 ± 1.0 mm, 17.6 ± 0.6 mm, and 16.3 ± 0.5 mm in light conditions at maximum Se-NPs concentration (400 µg mL^−1^) against *B. subtilis*, *S. aureus, P. aeruginosa*, and *E. coli*, respectively. In the current study, the antimicrobial activity was decreased by lowering the ZnO-NP concentration. For instance, at 100 µg mL^−1^, the inhibition zones were 15.4 ± 0.6, 14.4 ± 0.6, 20.7 ± 0.7, 16.7 ± 0.6, and 15.3 ± 0.6 mm toward pathogens of *B. subtilis*, *S. aureus*, *P. aeruginosa*, *E. coli*, and *C. albicans*, respectively (Figure 6). 

The lowest active ingredient concentration that effectively inhibits the development of microorganisms is known as the minimum inhibitory concentration (MIC). The type of microorganisms and active substances affect this value [68]. Before using the produced active compounds in biomedical and pharmaceutical uses, it is crucial to determine the MIC value. Because of this, the activity of various concentrations of synthetic ZnO-NPs was examined. Data analysis showed that the MIC value was 12.5 µg mL^−1^ for *B. subtilis* (9.3 ± 0.6 mm) and *C. albicans* (9.7 ± 0.6 mm), whereas it was 6.25 µg mL^−1^ for *S. aureus* (8.5 ± 0.5 mm), *P. aeruginosa* (10.7 ± 0.6 mm), and *E. coli* (8.7 ± 0.6 mm). The green synthesized ZnO-NPs showed antibacterial activity against *E. coli* and *S. aureus,* with MIC values of 50 and 100 µg mL^−1^, which are high as compared with the value in the current study [73], indicating the high activity of the current ZnO-NPs. As shown in Figure 6, the phyto-synthesized ZnO-NPs were highly active against *P. aeruginosa,* as indicated by high inhibition zones and lowering MIC values, followed by *S. aureus*, *E. coli*, and *C. albicans* in light conditions. 

The bacterial cell wall has a negative charge due to the presence of peptidoglycan, which has amino and hydroxyl groups that are negatively charged. Additionally, the presence of teichoic acid is enriched with phosphate components that have negative charge [74]. The zeta potential analysis confirms the presence of positive charge on the surface of synthesized ZnO-NPs. Therefore, the electrostatic attraction between ZnO-NPs (positive charge) and negative charge of bacterial cell wall was achieved.

As shown in Figure 6, the Gram-negative bacteria were more sensitive toward plant-based ZnO-NPs compared to Gram-positive bacteria. This is attributed to the cell wall structure, which, in Gram-positive bacteria, contain a thick layer of peptidoglycan that can retard the penetration of ZnO-NPs [75]. Moreover, the Gram-negative bacteria contain high amounts of lipopolysaccharide that bear a negative charge, which is electrostatically attractive to positively charged NPs [56,76]. Among tested bacterial strains, *P. aeruginosa* was more sensitive to phyto-synthesized ZnO-NPs, followed by *E. coli*, *B. subtilis*, and *S. aureus.* The activity of ZnO-NPs against *C. albicans* could be related to destroying the sterol profile, which is important for rigidity, homogeneity, and integrity of the plasma membrane [77,78]. Additionally, the accumulation of ZnO in the *Candida* cell wall destroys sulfur-containing amino acids, such as methionine and cysteine [79]. 

The distinctive inhibitory mechanisms of ZnO-NPs may be due to their ability to damage bacterial cell wall integrity, generating toxic ions (Zn^2+^) inside the microbial cells, as well as producing high levels of reactive oxygen species (ROS) [30,80]. The uncontrollable release of ions and key components from the outside to the inside and vice versa is caused by the direct interaction of bacterial cells (which have negative charge) and ZnO-NPs (which have positive charge, according to the ζ-potential). This results in membrane dysfunction, the destruction of cellular activities, disruption of selective permeability, and membrane dysfunction [56,81]. The attraction of ZnO-NPs toward bacterial cells could be related to the electrostatic force between negatively charged bacteria and positively charged NPs [30]. In addition, the destruction of the bacterial cell walls could be attributed to the abrasive surface texture of ZnO-NPs, which are known as surface defects, such as surface chemistry, edges, and corners. These surface defects have a main negative impact on the mechanical damage caused to the bacterial cell walls by ZnO-NPs that lead to cell death [82]. To confirm the aforementioned mechanism, Ramani et al. showed that the antibacterial activity of nano-ZnO is dependent on surface defects, whose subsequences are shape-dependent [83]. The release of poisonous ions (Zn^2+^) into the surrounding environment, which contains bacteria and nanostructure, is another potential inhibitory mechanism of ZnO-NPs [84]. The main target of these toxic ions is the inhibition of the active transport system and disruption of enzymes and amino acids metabolism [85]. The release of these toxic ions can be affected by the characteristic features of synthesized NPs, such as concentrations, porosity, morphology, and size. Additionally, environmental conditions, such as light illumination, pH, media components, and exposure times, can affect the liberation of Zn^2+^ [86]. Peng and co-authors reported that the generation of Zn^2+^ is highest in spherical shape with smaller sizes than nano-rod structure with larger size because of high equilibrium solubility [87]. The aforementioned phenomenon can be employed in the current study as a valid explanation for the significant antibacterial activity of photosynthesized ZnO-NPs with spherical shapes and tiny sizes (10–45 nm).

The third inhibitory mechanism of ZnO-NPs is the generation of ROS, which has high oxidizing and reactivity properties. Among these reactive species are H_2_O_2_ (hydrogen peroxide), O_2_^•–^ (superoxide radicals), and ^•^OH (hydroxyl radicals), which enhanced their activity [30]. Because of the negative charge of O_2_^•–^ and ^•^OH, it cannot penetrate the bacterial cell membrane, and their action was on the bacterial outer surface, whereas H_2_O_2_ passes the bacterial cell wall, causing cell death [88]. The generation of ROS is increased with smaller sizes of ZnO-NP, as mentioned previously by Padmavathy and Vijayaraghavan [82]. The authors investigated the bactericidal efficacy of three varied sizes, 12 nm, 45 nm, and 2 µm, and they showed that the toxicity was increased with size 12 nm compared with other sizes, and they attributed this feature to the high secretion of ROS under irradiation (UV and visible lights). 

### 3.4. Catalytic Activity

**Figure 7:**
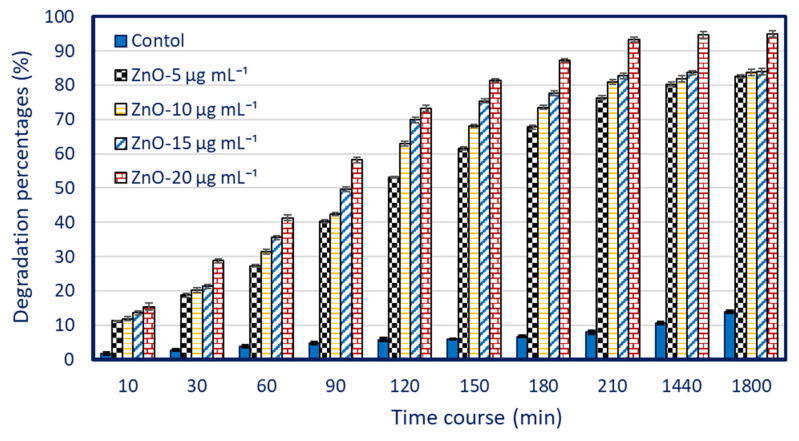
Photocatalytic properties of various concentrations (5, 10, 15, and 20 µg mL^−1^) of ZnO-NPs fabricated by peel aqueous extract of *P. granatum* to degrade MB. Data were represented by means ± SD (n = 3).

The catalytic properties of *P. granatum*-mediated green synthesis of ZnO-NPs were examined using methylene blue (MB) dye. The synthesized nanostructure was combined with MB solution (10 mg L^−1^), which was then transferred to the experiment’s settings after 30 min of dark incubation to reach the adsorption–desorption equilibrium. Figure 7 showed that the degradation percentages of MB under light conditions were correlated with nano-catalyst concentration and incubation time. Similarly, the activity of nanoflower-ZnO synthesized by leaf extract of *Calliandra haematocephala* to remove MB dye was increased with incubation times [89]. Additionally, the efficacy of two synthesized nano-catalysts, namely, MgO-NPs and α-Fe_2_O_3_-NPs, was observed to degrade real textile wastewater, which was concentration- and time-dependent [20]. The maximum dye removal rates that are correlated with high ZnO-NPs concentrations may be caused by the high numbers of active sites on nano-catalyst surfaces that rise with concentration or by the high creation of ROS that play a crucial role in dye degradations [90,91]. 

Data analysis showed that the removal percentages of MB dye under UV-irradiation conditions at ZnO-NPs concentration of 5 µg mL^−1^ was 11.2 ± 0.1% and increased gradually with incubation time to be 53.2 ± 0.3, 61.6 ± 0.5, 67.6 ± 0.6, and 76.3 ± 0.5% after 120, 150, 180, and 200 min., respectively. Interestingly, the decolorization percentages were increased with increasing ZnO-NPs nano-catalyst concentration. For instance, the dye removal percentages were increased from 76.3 ± 0.5% at a concentration of 5 µg mL^−1^ to reach 80.9 ± 0.7 and 82.8 ± 0.8% at concentrations of 10 and 15 µg mL^−1,^ respectively (Figure 7). Moreover, the highest dye degradation percentages of 93.4 ± 0.1% were attained after 210 min at maximum ZnO-NPs concentration (20 µg mL^−1^) under irradiation conditions (Figure 7B). Analysis of variance revealed that the catalytic activity in the removal of MB does not have significant difference at times of 210, 1440, and 1800 min at each separate concentration (Figure 7). This phenomenon could be explained by the sorption of dye on the adsorption sites on the nano-catalyst surface after 210 min. The obtained results were consistent with those obtained after 270 min of treatment with ZnO-NPs made from plant extract of *Calliandra haematocephala* [89]. Additionally, *Becium grandiflorum* leaf extract made ZnO-NPs exhibit the ability to degrade MB solution (100 ppm) with percentages of 69% after 200 min in the presence of UV irradiation [92].

**Figure 8:**
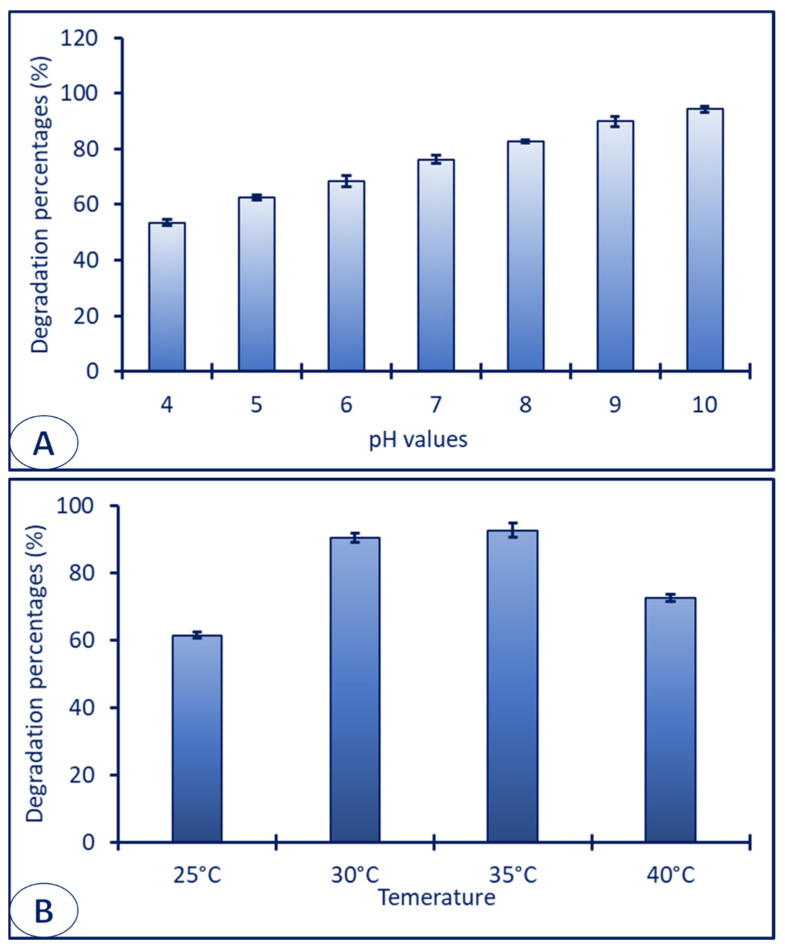
Effect of environmental factors, including different pH values (**A**) and different incubation temperature (**B**) on the sorption of MB after treatment with ZnO-NPs under optimum conditions.

The transformation of blue color to colorless can be attributed to the action of some physicochemical parameters, such as the pH of the treatment solution (halochromism) or the action of temperature (thermochromism) [93]. Therefore, the effect of different pH values and different incubation temperatures on the dye removal of MB were investigated under optimum conditions (20 µg mL^−1^ of ZnO-NPs for 210 min). Data analysis showed that the dye removal efficiency under UV-irradiation conditions was increased under alkaline conditions (Figure 8A). Data analysis showed that the UV enhanced dye removal under alkaline conditions. The decolorization percentages reached maximum values at pH 10, with a percentage of 94.3 ± 1.1% under UV-irradiation conditions (Figure 8A). In a similar study, the high removal of MB dye after treatment with ZnO-NPs under UV-light was attained at alkaline conditions (pH = 10) [94]. Erats et al. [95] reported that the pH values have a significant role in sorption of dyes by different catalysts because the pH can change its surface charge. For instance, the surface charge of a catalyst becomes positive and negative at acidic and alkaline conditions, respectively. Therefore, the sorption of cationic dye was increased at high pH and decreased at alkaline conditions. Moreover, Chanu et al. [94] reported that the removal potential of MB dye by ZnO-NPs was decreased at acidic conditions due to the dissolution of ZnO at low pH. Additionally, the authors reported that the pH = 9 is the zero-point charge of ZnO-NPs and, above this value, the surface of ZnO bears negative charge, which results in it being attracted toward the positive charge of MB in an aqueous solution.

Temperature is another factor affecting the decolorization and sorption of dyes on the surface of ZnO-NPs. Data analysis showed that 35 °C is the best temperature degree for dye removal, with percentages of 92.7 ± 2.1% under UV-emission, respectively (Figure 8B). The sorption or dye removal was decreased when the temperature degree increased or decreased more than 35 °C. 

**Figure 9:**
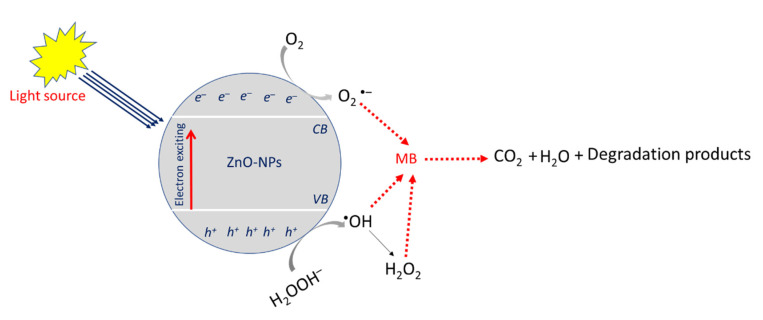
Photocatalytic mechanism of ZnO-NPs for the degradation of MB under UV-irradiation conditions.

The enhancement of dye removal in the presence of UV-irradiation (photocatalysis) could be due to its effects on the exciting of the electrons from the valance band (VB) to the conductance band (CB) (Figure 9). Once light photons are absorbed by the surface of ZnO-NPs, the excited electrons (e^−^) migrate from the valence band to the conductance band, leaving holes (h^+^) there (Equation (3)). The reduction process was achieved on the CB, forming a superoxide anion (^•^O_2_^−^) via interaction of excited e^−^ with O_2_ (Equation (4)). The as-formed O_2_^−^ interacts with H^+^ to form hydrogen peroxide (HO_2_^•^), which reacts with dissolved H^+^ to generate hydrogen peroxide (H_2_O_2_) (Equations (5) and (6)). On the other hand, the oxidation process was achieved in VB through reaction of h^+^ with H_2_O, forming highly oxidizing hydroxyl radicals (^•^OH) (Equations (7)). Finally, the forming various radicals started to attack the MB dye and degrade it to less harmful compounds, H_2_O and CO_2_ (Equations (8)) [5,30].

(3) $$\mathrm{UV}-\mathrm{irradiation}+\mathrm{ZnO}-\mathrm{NPs}\to e^{-}+h^{+}$$

(4) $$e^{-}+O_{2}\to^{\cdot }O_{2}{}^{-}$$

(5) $$O_{2}{}^{-}+{\;H}^{+}\to {\;\mathrm{HO}}_{2}{}^{\cdot }$$

(6) $$HO_{2}{}^{\cdot }+H^{+}\to H_{2}O_{2}$$

(7) $$h^{+}+H_{2}O\to HO^{-}$$

(8) $$\mathrm{MB}+\mathrm{radicals}{(}^{•}O_{2}{}^{-},H_{2}O_{2},HO^{-})\to \mathrm{less}\;\mathrm{harmful}\;\mathrm{compounds}+CO_{2}+H_{2}O$$

**Figure 10:**
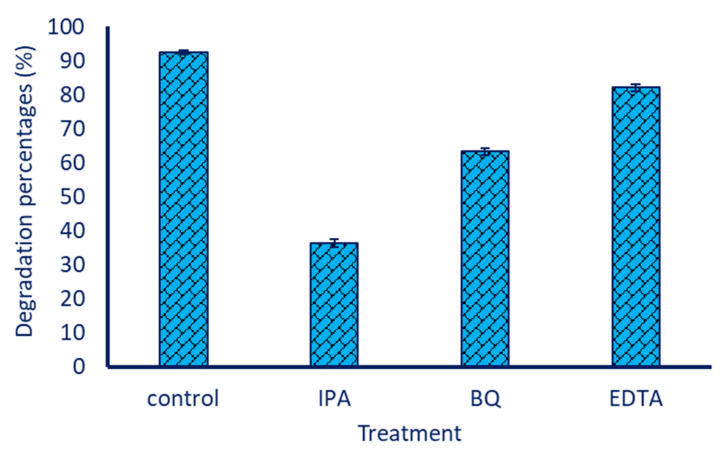
Effect of different scavengers, including isopropyl alcohol (IPA), benzoquinone (QB), and ethylenediaminetetraacetate (EDTA) on degradation percentages of MB using green synthesized ZnO-NPs.

The role of various active species in photocatalysis and degradation of MB was measured using the trapping method. As shown in Figure 10, the MB degradation percentages were decreased after the addition of different free radical scavengers of IPA, QB, and EDTA to the photocatalytic experiment. Result analysis revealed that the presence of various free radical scavengers reduced the efficacy of MB degradation by ZnO-NPs. As shown, the maximum decrease in MB degradation percentage (36.4 ± 1.2%) was attained due to the presence of IPA, which captured ^•^OH. Moreover, after the addition of QB and EDTA, the degradation percentages were reduced to 63.3 ± 0.9% and 82.1 ± 1.0%, respectively, which captured ^•^O_2_^–^ and h^+^. In the same regard, the degradation percentages of ciprofloxacin, using green synthesized ZnO-NPs under UV-irradiation, were significantly decreased after the addition of scavengers IPA, QB, and EDTA [29]. According to these results, the addition of IPA led to the greatest decline in degradation percentages, which was then followed by the addition of EDTA and BQ. Based on the obtained data, it can be concluded that the ^•^OH has a significant role in photocatalytic MB degradation, followed by ^•^O_2_^−^ and h^+^. 

### 3.5. Sorption of Dye on the Surface of ZnO-NPs

**Figure 11:**
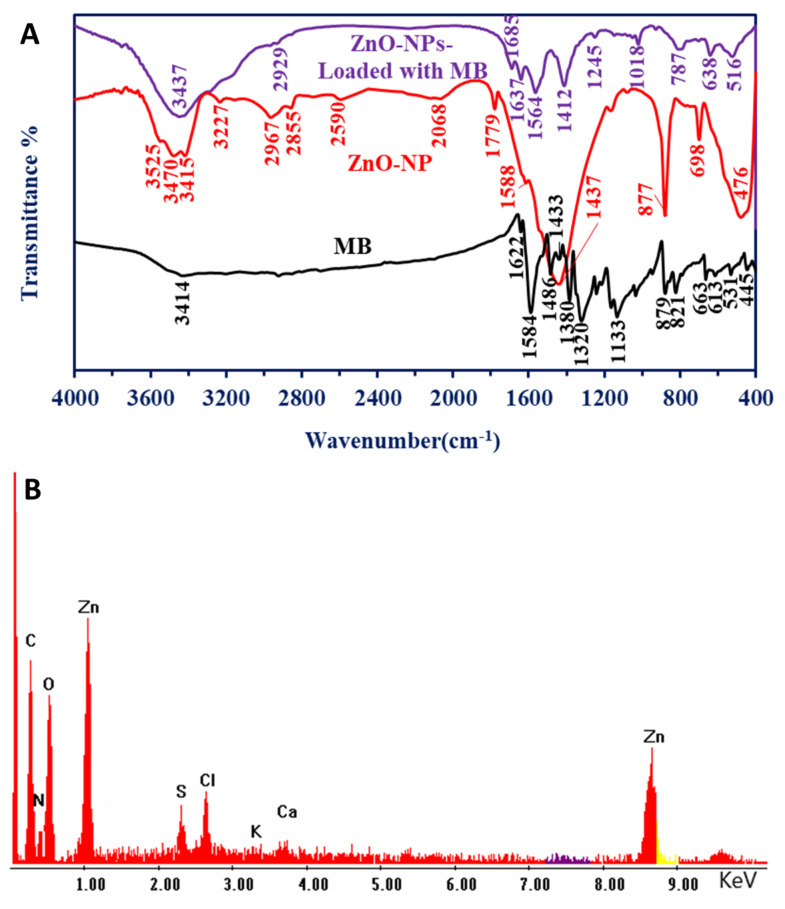
The FT-IR (**A**) and EDX chart (**B**) of phyto-synthesized ZnO-NPs after reacting with MB dye under optimum catalytic conditions (20 µg mL^−1^ of nano-catalyst after 210 min under light emission conditions).

Due to time being a crucial factor to be applied to the nano-catalyst on large scale, 210 min was selected as the optimum time for degradation of MB under visible light conditions at a ZnO-NPs concentration of 20 µg mL^−1^. The nano-catalyst was collected and examined under ideal conditions to confirm the sorption of MB dye on their surface by FT-IR and SEM-EDX, based on the aforementioned finding. As shown, due to the adsorption or decomposition of MB on the ZnO-NPs (Figure 11A), the spectra were changed accordingly. Figure 11A exhibited the comparison between FT-IR spectra of MB and ZnO-NPs, and these were recorded after being loaded with MB. The broadness, related to NH and OH, has appeared with high intensities for ZnO-NPs compared to MB (which appeared at 3414 cm^−1^ as low intensity), and this is related to the functional groups of carbohydrate moiety and MB substrate that appeared at 3437 cm^−1^. Other significant peaks of MB appeared at 1622 and 1584 cm^−1^, which are related to the C=N of amide, C=C aromatic, and NH groups. A peak at 1486 cm^−1^ was related to C=C-C of aromatic, in which the peak at 1433 cm^−1^ of C-H bend. Peaks at 1380, 1320, and 1133 cm^−1^ were related to the stretch C-N of aromatic tertiary amine in the MB structure. Peaks at 879 and 821 for C-S stretching were observed. The figure-print peaks were from 663 to 445 cm^−1^ for C-C, C-H, and Cl bending. In the spectra of the ZnO-NPs after reacting with MB, the peak at 2929 cm^−1^ corresponded to CH aliphatic. The peaks related to some groups, such as SH and S=O, disappeared. Additionally, peaks at 1685, 1637, and 1464 cm^−1^ were noticed, and they corresponded to C=O and NH groups [96]. Decreasing the intensity of the peak at 1437 cm^−1^ satisfied the sulfate groups and resulted in a shift to 1412 cm^−1^, as well as another low-intensity peak at 1245 cm^−1^ for C-N of the tertiary and quaternary amines of the MB moiety [51]. Peaks at 1018 cm^−1^ correspond to the C-N stretching and C-O-C asymmetric stretching. Moreover, peaks at 787 cm^−1^ (a new peak) are related to C-S stretching, and a peak at 638 cm^−1^ (shifts from 698 cm^−1^ of the original material) signified that the OH that is out-of-plane overlapped with polysulfides [52]. Shifts of the peak from 476 cm^−1^ to 516 cm^−1^ are related to the sulfide derivatives used in the adsorption processes [97], as shown in EDX analysis. 

The EDX analysis for ZnO-NPs, following MB loading, revealed the presence of new peaks, as well as changes in the weight and atomic percentages of the peaks that were already present in the original chart, showing the sorption of dye on the surface of the nano-catalyst (Figure 11B). The presence of new peaks, such as S with weight and atomic percentages of 2.07% and 1.25%, could arise from MB decomposition (Figure 11B). Moreover, an increase in the weight and atomic percentages of specific peaks, such as C and N, as shown in Figure 4D in comparison to Figure 11B, is another indicator that MB has adhered to the surface of the nano-catalyst. In a recent study, the EDX chart of Se-NPs, after reacting with MB, showed that the presence of some peaks, such as S and N, as well as an increase in the weight and atomic percentages of some already existing peaks, such as C and Cl, confirm successful decomposition [72]. 

### 3.6. Reusability of Nano-Catalyst

**Figure 12:**
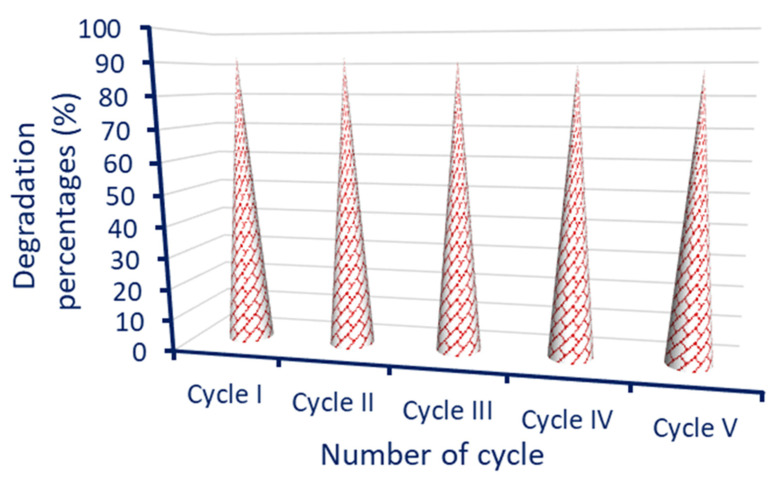
Reusability of nano-catalyst ZnO fabricated by aqueous peel extract of *P. granatum* under optimum conditions for five repeated cycles.

The investigation of the reusability or recyclability of ZnO-NPs is an important step to be applied on a large scale [27]; therefore, five cycles were conducted. The reusability test was assessed under optimum catalytic conditions, which are 20 µg mL^−1^ of ZnO-NP concentration under UV-irradiation conditions for 210 min. The ZnO-NPs were collected at the end of each cycle and washed thrice with distilled H_2_O and oven dried at 60 °C before being added to the next cycle. The obtained data reveal the outstanding stability of ZnO-NPs in the degradation of MB for five cycles with a limited decrease in their catalytic activity (Figure 12). Data analysis showed that, by repeating the photo-catalytic cycles, the photodegradation of MB after the first cycle was achieved with percentages of 92.3 ± 0.2% with a little decrease. As shown in Figure 12, the degradation percentages after the second cycle were 91.7 ± 0.3% and reached 88.2 ± 0.3% after the fifth cycle. The decreasing photocatalyst activity of nano-catalysts was recorded by various researchers after reuse. Data reported by Algarni et al. [98] showed that the activity of ZnO-NPs fabricated by leaf aqueous extract of Rosmarinus officinalis to degrade MB and crystal violet was decreased from 99.9% and 99.6% at the first cycle to 92.5% and 91.2% after the third cycle, with decreasing percentages, ranging from between 7% to 8%. Similarly, the catalytic activity of ZnO-NPs, CuO-NPs, and ZnO/CuO nanocomposites, during MB degradation under light conditions, was highly stable, even in ten repeated cycles with a 4% decrease in their activity [99]. 

## 4. Conclusions

Herein, green synthesis of ZnO-NPs was achieved using an aqueous extract of *P. granatum* peels as a biocatalyst for reduction and capping/stabilizing of the final product. The physicochemical characterization was accomplished by UV-Vis spectroscopy (showing maximum SPR at 345 nm), FT-IR (exhibiting the role of various function groups in plant extract in reduction and stabilizing ZnO-NPs), XRD, SAED (improving the crystalline nature), TEM (showing the size of biosynthesized ZnO-NPs was in the range of 10–45 nm with an average of 25.1 nm), and SEM-EDX (confirmed the maximum components of synthesized NPs were Zn and O with weight percentages of 55.29 and 24.34%, respectively). The synthesized ZnO-NPs exhibit antimicrobial and catalytic activities in a dose-dependent manner. Data analysis showed that the Gram+, Gram-, and unicellular fungi were sensitive toward ZnO-NPs at low concentrations, with MIC values of 12.5–6.25 µg mL^−1^. Interestingly, the *P. granatum*-based ZnO-NPs exhibited high catalytic activity through removing MB dye under UV-irradiation. The maximum dye removal percentages were 93.4 ± 0.2% at a concentration of 20 µg mL^−1^ after a contact time of 210 min in the presence of UV. Moreover, the nano-catalyst of ZnO-NPs is more stable for five repeated cycles with low decreasing activity. The current study confirms the hypothesis that the activity of ZnO-NPs was improved in the presence of UV-light irradiation. Overall, the aqueous extract of *P. granatum* peels, containing various metabolites, has efficacy to form ZnO-NPs with small sizes, which showed promising antimicrobial activity against a wide range of pathogens. Additionally, the synthesized NPs showed high dye removal activity under UV-light emissions. The obtained data open the way to integrate green synthesized ZnO-NP into biomedical and biotechnological sectors as an eco-friendly catalyst. 

## Data Availability

The data presented in this study are available upon request from the corresponding author.
